# Propofol’s Effects on the Fetal Brain for Non-Obstetric Surgery

**DOI:** 10.3390/brainsci7080107

**Published:** 2017-08-18

**Authors:** Ajay Malhotra, Emily Yosh, Ming Xiong

**Affiliations:** Department of Anesthesiology, New Jersey Medical School, Rutgers University, Newark, NJ 07107, USA; Ajaymal@gmail.com (A.M.); emilyyosh@gmail.com (E.Y.)

**Keywords:** Propofol, neurotoxicity, non-obstetric surgery, fetal brain development

## Abstract

While the use of Propofol has been increasing in usage for general surgical procedures since its release to market, there has been little work done on its potential link to neurotoxicity in humans. Only recently, following the release of a warning label from the United States Food and Drug Administration (USFDA) regarding a potential link to “neurotoxicity” in the neonate, did the surgical and anesthesiology communities become more aware of its potential for harm. Given the widespread use of this drug in clinical practice, the warning label naturally raised controversy regarding intrapartum Propofol usage. While intended to generate further studies, the lack of a viable anesthetic alternative raises issues regarding its current usage for surgical procedures in pregnant women. To answer the question whether current evidence is supportive of Propofol usage at its current levels in pregnant women, this review summarizes available evidence of fetal Propofol exposure in animal studies.

## 1. Introduction

Propofol was originally discovered in 1976 in the Biology Department of Imperial Chemical Industries (ICI) Pharmaceuticals (UK) amidst the development of over 5000 compounds intended as potential new anesthetics for clinical use. In 1977, clinical trials began on the usage of Propofol and the original formulation was withdrawn from the market due to the development of anaphylactic reactions in a subset of patients. This formulation was quickly replaced by a soybean oil/Propofol emulsion and re-launched in 1986 in its current form as Diprivan [1]. Propofol is an alkylphenol that quickly gained favor due to its rapid onset and offset, as well as its unique antiemetic effect. While the mechanism of action has not been fully elucidated, at low doses its enhancement of γ-aminobutyric acid (GABA)-induced chloride channels potentiates GABA activation via an indirect effect; at a higher dosage it is thought that Propofol functions directly on the GABA receptor [2].

Recently, and without warning, the United States Food and Drug Administration (USFDA) released a warning label regarding the use of Propofol in the neonate as it pertains to a potential for neurotoxicity [3]. According to the warning label released on 14 December 2016, a single brief exposure to general anesthetic drugs “is unlikely to have negative effects on behavior or learning”, but repeated or lengthy use of drugs like Propofol may have negative effects on brain development in children under the age of three or during fetal development, specifically in the third trimester. From the available evidence, it appears that much of this is based upon studies conducted in pregnant animals, young animals, and the sparse clinical studies done in children. Notably, this concern is not unique to Propofol; many anesthetic drugs have been shown to present somewhat of a toxic risk and are considered equally risky to the fetus and young children.

The use of Propofol in surgical practice is incredibly common in the induction and maintenance of anesthesia. While many patients appear to remain unaffected, the neonatal population may be vulnerable to associated toxic effects. Given that approximately 2% of pregnant women receive Propofol for non-obstetric surgical procedures in North America alone, many neonates, infants, and children are potentially exposed subjects.

## 2. *In Vitro* Studies

The possibility of Propofol-induced neurotoxicity has been studied in various animal models for more than a decade. Since El-Beheiry et al. [4] discussed the association of growth cone collapse and neurite destruction in *in vitro* samples of dorsal root ganglia, retinal ganglion cell layers, and sympathetic ganglion chains isolated from chick embryos, there has been significant interest in Propofol’s effects on neural cell function. Multiple different mechanisms have been found to be associated with Propofol exposure-induced cellular death, including oxidative DNA damage with halted cell proliferation [5], induced apoptosis of immature neurons [6], altered neurogenesis [7], inflammation in brain tissue [8], mitochondrial fission with dysregulation [9], epigenetic dysregulation (MicroRNAs) [10], and dysregulation of neurotrophin expression [11]. Goto et al. [12] demonstrated several changes in miRNA expression levels of the rat hippocampus induced by both sevoflurane and Propofol that were distinct from the control group. The most convincing mechanism associated with this topic is discussed in a paper by Wise-Faberowski et al. [13], wherein they also state that the immature brain is different from the brain of the adult in the neuronal composition.

Immature neurons need a more suitable environment with elements such as growth factors that support and form synapses to be functionally intact. Many of those benefits could be disrupted by exposure of the young brain to Propofol. Interestingly, there are differences between the mature and immature neurons in response to Propofol despite the mechanistic association of binding to the GABA-A receptor. According to Chen et al. [14], chloride ion influx causes inhibition in an adult neuron, and chloride ion efflux in an immature neuron leads to calcium ion influx and inappropriate cell excitation. While there are still questions as to whether those differences are truly able to explain the mechanism of Propofol neurotoxicity, it appears that Propofol triggers an internally harmful response from the immature neuron.

## 3. Animal Models

Much of the groundwork on the toxicity of Propofol has come about from work done in rodent models focused on a narrow age range of the animal’s neural development. In a 2008 study performed by Cattano et al. [15], a single intraperitoneal dose of Propofol was given, ranging from subanesthetic to a high dose (25–300 mg/kg), in young (5–7-day-old) mice. Despite the dosage required to achieve anesthetic levels being in the mid to high range (150–200 mg/kg), neuroapoptosis in the cortex and the caudate/putamen occurred in these mice at subanesthetic levels (at doses of 50 mg/kg). A seemingly isolated finding in the lab, this data was somewhat alarming for the prospect that sub-anesthetic levels of Propofol demonstrated a notable toxic effect on the brain. From there, subsequent work was done on rhesus monkeys to study Propofol’s effects on the developing brain and to provide contextual clues about what might be occurring in the human brain. Brambrink et al. [16] found increased levels of apoptosis following 5 h of continuous Propofol infusion of 250–350 mg/kg/min. Specifically, comparing the developing brains of 120 day gestational age rhesus monkeys to that of post-natal day 6 (P6) rhesus monkeys, the authors noted that postnatal exposure to Propofol targets neurons of the cerebral cortex. However, gestational Propofol exposure affects more the caudal and rostral regions of the brain (the regions characterized by the cerebellum, inferior colliculus, caudate, putamen, nucleus accumbens, amygdala, and thalamus). Interestingly, the study also found a correlative increase in oligodendrocyte death amongst the subpopulation beginning myelin generation. Following this, other studies have focused on differing dosages, durations of exposure, and locations/regions of neurotoxicity, which have elucidated increased cellular death ranging from causes such as the upregulation of caspase-induced apoptosis [17], to increased tumor necrosis factor-α (TNFα) release [7], to decreased dendritic spine density [18]. Taken together, there is compelling evidence of some degree of induced cellular death in the developing brain that is correlative to a notable change in cellular function. However, the question that still remains is whether this fosters a demonstrable phenotypic change in the offspring.

In the Xiong laboratory, ongoing studies have focused on evidence of neurotoxicity associated with prenatal exposure of Propofol in rats. Like several other animal and human embryonic stem (h-ES) cell studies, we believe that neuronal cell death could happen as early as the “fetal stage”. Also, to follow up on recent animal studies on Propofol neurotoxicity and understand potential correlations between Propofol-induced neuronal death and long-term brain development, we believe that Propofol can be implicated in the induction of necrosis/apoptosis of the immature neuron. However, it appears that the long-term effects of this neuronal death on overall brain development may not play as substantial role as initially feared [19].

In 2014, Xiong et al. [17] specifically analyzed the effect of an anesthetic dose of Propofol via infusion at 0.4 mg/kg/min for 2 h in pregnant rats on gestational day 18. The rats were broken into two groups, one of which was subjected to C-section and the other to normal spontaneous delivery, which usually occurred 48–72 h after administration. The authors analyzed both molecular and behavioral outcomes following Propofol administration. The findings suggest that the Propofol-treated group showed significantly less exploratory activity in an open-field test, less spatial learning, and persistent learning deficits compared to untreated controls. Furthermore, it appears that Propofol exposure during pregnancy increased apoptotic effects in the fetal brain (elevating caspsase-3 levels), increased deletion of neurons, and reduced synaptophysin levels in the hippocampal region of rodents. The findings of this paper seem to support the theory that the actual phenotypic effect associated with neuronal cell death as seen in this patient population is transient, not permanent. The proposed explanation for this appears to be that while actual neuronal cell death does occur secondary to Propofol exposure, the sheer neural cell volume is so abundant that even with toxicity-associated apoptosis, the residual neuronal volume is able to compensate for the cell loss without any lasting effects.

## 4. Clinical Studies

The clinical studies performed have been less clear, but some have suggested an association between early exposure to anesthesia drugs and neurodevelopmental delay. An early paper by Flick et al. [20] in pediatrics examined cognitive/behavioral outcomes in early exposure to anesthesia and surgery. While correlative, a retrospective review such as this contains far too many variables to draw definitive conclusions regarding the isolated use of Propofol and its potential for behavioral modifying neurotoxicity. Chief among such problems are that pediatric patients requiring surgery at such a young age are already suffering from comorbid conditions which may affect behavior and development. Further confounding factors in such studies are that patients requiring surgical interventions require a balanced anesthetic approach, thereby necessitating multiple medications throughout surgery and well into the perioperative period. With no real way to conduct such a sham surgery without a gross breach of ethical decorum, it is impossible to isolate the effects of Propofol alone on this patient population.

The most recent studies published concerning clinical trials in human subjects are the General Anesthesia Compared to Spinal Anesthesia (GAS) trial [21] and the Pediatric Anesthesia Neurodevelopment Assessment (PANDA) [22]. Not strictly dealing with the issue of intraoperative Propofol use, the GAS trial is an ongoing international, multicenter, randomized controlled prospective trial focused on outcomes in children younger than 60 weeks, but born at more than 26 weeks gestation, requiring inguinal hernia repair. The focus is on the use of sevoflurane in this patient population, with the primary outcomes of the trial on neurocognitive development in patients receiving awake-regional anesthesia versus sevoflurane-based general anesthesia. The primary outcome of the trial uses the Wechsler Preschool and Primary Scale of Intelligence Third Edition (WPPSI-III) Full Scale Intelligence Quotient (IQ) at the age of 5 years. An interim result using the secondary outcome of the Bayley Scales of Infant and Toddler Development III at the age of 2 years was built into the trial. In January 2016, the two-year follow-up result was published, and available data from 238 children treated with awake-regional anesthesia versus 294 children provided general anesthesia was analyzed. In the sevoflurane administered group, the median duration of general anesthesia was 54 min. Per the secondary outcome result, the authors reported no difference in Bayley III development scores between the two study arms and suggested that the data support the conclusion that sevoflurane anesthesia of less than 1 h duration does not appear to increase the risk of adverse neurodevelopmental outcome at the age of 2 years when compared to awake-regional anesthesia. The GAS study is still ongoing and pending the primary WPPSI-III IQ outcome measure at the age of 5 years. While the methodology of the trial focused on sevoflurane-based induction, a negative result from this result may prove promising for enabling future studies with the concomitant use of Propofol and sevoflurane, given the exclusionary result that a sevoflurane only induction would provide.

The PANDA study is a sibling-matched observational cohort study which examined a single anesthesia exposure in healthy children younger than 3 years of age. The focus of this study was a measure of impaired global cognitive function (IQ) as the primary outcome. The secondary outcome was an assessment of abnormal domain-specific neurocognitive functions and behavior at ages 8 to 15 years. A cohort of exposed children (*n* = 105) were provided with general anesthesia for elective inguinal hernia surgery. The children were under 3 years old, at least 36 weeks gestational age or older at birth, and approximately 90% of the subjects were male. In the exposed group, the average anesthetic duration was 84 min (17 children had exposures of greater than 2 h). The unexposed cohort (*n* = 105) were biologically related siblings that were within 3 years of age to the exposed child, aged 36 weeks gestational age or greater at birth, and had not had any anesthesia exposure prior to 3 years of age. The study findings were that that mean IQ scores were not significantly different between the exposed and unexposed siblings (both groups scored somewhat higher than average). Thus, neither trial definitively linked the use of anesthetics with any form of developmental delay. Both trials also demonstrated that brief exposures to general anesthesia in an otherwise healthy child did not seem to cause any overt defects. Both studies are still underway, and primary outcomes data are required to make more definitive determinations about if and/or when early exposures to anesthetics may be deemed problematic.

In 2015, Chidambaran et al [23] detailed the use of Propofol in pediatric cases, a common practice in hospitals across the US, but still considered an off-label use in children under the age of 3 years. The authors found that, despite advantages over inhalation anesthesia, including reduced post-operative nausea and emergence delirium, there are several potential dangers that need to be explored. Specifically cited was the fact that Propofol functions as an uncoupling agent in oxidative phosphorylation and has implications in children with underlying mitochondrial disorders, creating the potential for life-threatening complications.

In January 2017, The American Academy of Pediatrics (AAP) weighed in, reiterating the content of the USFDA statement [24]. In a short statement, the Academy, along with a coordinated Section on Anesthesiology and Pain Medicine and the Committee on Drugs, stated that the FDA warning was not based on new information and had been discussed in previous FDA sections on the topic as early as 2007. Despite ongoing studies, they wanted the warning placed in “the perspective of recent controlled trials in humans and multiple epidemiological studies of large homogeneous populations … which demonstrate no developmental problems in children exposed to a single, short anesthetic or sedation”. Furthermore, they cautioned against the risks of delaying needed surgery and diagnostic procedures because of this warning, as additional information is required to accurately gauge “the risks and benefits of each contemplated procedure prior to proceeding”. The potential effects of Propofol on the fetal brain has become a concerning issue since it was identified as an agent potentially linked to neuronal death in the young brain.

## 5. Recent Studies

Despite the fact that clinical evidence does not support the contention that Propofol is linked to neurotoxicity, laboratory studies continue to report experimental data that support the converse [25,26,27]. In light of the fact that fetal exposure to Propofol in the immature neuron has been seen in multiple animal studies, it may not be disputed that Propofol may have an association with increased neuronal cell death. Much of the work that has been done thus far has been focused on in vivo rat studies assessing the short-term outcome of Propofol and increased cellular toxicity or decreased neurogenesis. However, the ongoing clinical studies highlighted in this paper are limited in scope to the use of inhaled anesthetics, and interim results appear to demonstrate minimal phenotypic changes as a result of inhaled anesthetic agents. The question that arises now is what might the effects of Propofol be on long-term patient outcomes and what may be done to mitigate such untoward effects?

In 2016, Jiang et al. [28] published a study that analyzed the effects of inhaled anesthetic via the long-term fate mapping of neuronal growth in transgenic mice, and found that hippocampal neurons were damaged following isoflurane exposure. The affected populations demonstrated a significant amount of increased apoptosis noted in the hippocampal cell population but, notably, at two weeks and two months, rates of neurogenesis were equivalent among the control and exposed population with significant recovery of neural maturation. While this too is not directly correlative to a direct Propofol exposure study, it provides solid evidence that “anesthetic-induced apoptosis” may not be a lasting effect. Adjacent cellular productivity can increase, thereby compensating for loss induced secondary to volatile general anesthetics. However, it is less clear whether apoptotic activity and Propofol-induced loss would share the similar fate.

Through investigating the single versus multiple dosing model, Chen et al. [29] concluded that apoptosis or synaptic loss can be induced by a single or by multiple doses of Propofol in neonatal rats, but long-term neuronal damage was only verified in a multiple exposures model and not in the single exposure model. Those results strongly indicate that the long-term cognitive dysfunction seen in neonatal rats was actually due to multiple exposures of Propofol, while minimal damage was seen in a single exposure. While there does not appear to be data behind the rationale for this treatment modality yet, the thought process appears to be that increased exposure to Propofol increases the brain’s neurotoxic exposure. Thus, in the single exposure model, it is thought that a single dosing of Propofol provides a “hit” to the brain. Taking this rationale further, it appears to indicate that increased Propofol exposure through multiple doses increases the toxicity to the brain, thereby increasing cellular death.

Another potentially interesting point in this field of study may be the neuroprotective effect that different compounds may be able to provide, thus mitigating Propofol’s neurotoxicity. Noticeable agents which have been used in rodent studies have included the administration of an antioxidant (i.e., circumin) [30], lithium [31], erythropoietin [32], and dexmedetomidine [33]. In particular, Dexmedetomidine (Dex)-pretreated rodent neuronal cultures exposed to Propofol appear to have attenuated hippocampal neurotoxicity as compared to controls. Notably, results showed that while Propofol exposure alone reduced cellular viability, induced apoptosis, and decreased protein expression of cytoprotective proteins, Dex-pretreated cells had an attenuated effect to Propofol-induced neuronal apoptosis and also had increased cytoprotective protein expression. While studies suggest that Propofol-induced apoptosis is an oft-observed phenomenon, it appears to be preventable, potentially reversible, and without a long-lasting effect on neural development [34].

It remains a challenge to answer the question as to whether direct fetal Propofol exposure during pregnancy has similar outcomes (single dose versus multiple dose) in rat studies. From the GAS and PANDA studies, it appears that fetal exposures to anesthetics with a demonstrable effect in the rodent population do not appear to affect human subjects with the same level of neurocognitive dysfunction. While primary outcomes are still pending, it is uncertain whether the human population is simply more resilient and capable of demonstrating a “bounce-back” effect from the neurotoxic exposure of anesthetic as opposed to the rodent population. Simply put, the sheer number of neuronal loss may be compensated for by the fact that full pruning of the human neural network may not have occurred at the time of neural insult. Thus, the sheer cellular volume may be capable of compensation for Propofol’s toxic effects.

It is important to distinguish which groups exist in those patients that require surgical intervention. On one hand, a set of patients may require one surgical intervention in isolation versus multiple interventions throughout a pregnancy. These patients may either necessitate multiple surgical interventions receiving induction dosages each time, or they may require further intervention and receive either Propofol infusions or multiple doses of Propofol intraoperatively. Another overarching group may require a continuous infusion (i.e., total IV anesthesia) versus a single induction dose. In these cohorts, the infusion patients are allowed to receive high levels of Propofol such that it may approach a steady-state intracranially in the neonate, thus maintaining a greater degree of toxicity. Within any of these patient populations, the developing neonate may receive “one hit” alone, “one hit” multiple times, or “multiple hits” regarding toxicity to the developing brain. However, patients that receive an induction dose alone may only take the “one hit” in terms of toxicity.

Acknowledging that the knowledge gap is problematic, we simply do not nor cannot know which of the above groups may mediate the greatest level of toxicity to the developing fetus. Perhaps, as Wilder et al. suggested [35], those patients that require multiple interventions are either receiving multiple toxic interventions and at increased risk for disability or they are already within a higher risk cohort that would go on to develop disability regardless of surgical intervention. Nonetheless, our goal is to provide a rubric for anesthetic approaches and raise the possibility that surgical intervention in pregnant women may best be approached by the use of regional anesthesia.

## 6. Conclusions and Current Recommendations

The FDA has issued its warning label out of an abundance of caution, especially in the demographic of pregnant females. The fact that single exposures of Propofol do not appear to have a viable negative long-term effect on a neonate’s developing brain in animal studies has not played into the issuance of the warning label is baffling. Long-lasting effects such as memory loss and behavioral changes have not been definitively proven in animal studies. Further confounding data is that the amount of Propofol and the duration of exposure may play a critical role in “Propofol-induced neurotoxicity”. Thus, the lack of convincing evidence of studies involving humans and potential Propofol neurotoxicity leads us to believe Propofol to be a tolerable anesthetic method for application in pregnant women for medical/ surgical needs. However, as seen in animal studies with Propofol usage, perhaps it is warranted that caution be used and avoidance of multiple dosing be sought in the parturient. Furthermore, we emphasize the fact that because “abnormal neuronal cell death” may happen in animal studies, a reduction in exposure time, dosage, avoidance of repetitive Propofol exposure, use in combination with Dexmedetomidine, avoidance of Propofol entirely, or opting for regional anesthesia during pregnancy may be prudent changes to the anesthetic plan for pregnant females.
